# Quality assessment in bone densitometry: a case of incorrect hip analysis

**DOI:** 10.1093/bjrcr/uaae038

**Published:** 2024-10-22

**Authors:** Hongmin Xu, Medhat Sam Gabriel, Judy Rose James

**Affiliations:** Department of Radiology and Medical Imaging, Loyola University Medical Center, Maywood, IL 60153, United States; Department of Radiology and Medical Imaging, Loyola University Medical Center, Maywood, IL 60153, United States; Department of Radiology and Medical Imaging, Loyola University Medical Center, Maywood, IL 60153, United States

**Keywords:** dual-energy X-ray absorptiometry (DXA), bone densitometry, quality assessment, osteoporosis

## Abstract

Dual-energy X-ray absorptiometry (DXA) is a sophisticated imaging technique utilized in the field of medical diagnostics to measure bone mineral density. The significance of DXA lies in its ability to accurately assess bone health, which is crucial in the evaluation of osteoporosis, a condition characterized by weakened bones and heightened susceptibility to fractures. Despite its widespread adoption and clinical utility, DXA is not without limitations. Factors such as body size, tissue hydration, the presence of metal implants, improper equipment installation and maintenance, as well as inadequate education and training in bone densitometry may influence DXA measurements, necessitating careful interpretation by trained professionals. We present a case to show the errors that occurred during DXA analysis leading to dramatic T-score changes, highlighting the importance of technologists’ adherence to manufacturers’ recommendations, to ensure the accurate processing of DXA scans and diagnosis.

## Clinical presentation and imaging findings

A 60-year-old female presented for a follow-up evaluation of osteoporosis. Upon initial dual-energy X-ray absorptiometry (DXA) scan, the report indicated a right femoral neck T-score of -1.6, which showed minimal deviation from the previous year’s result of -1.4. However, the left femoral neck T-score was reported as -6.8, which was markedly abnormal and exhibits a significant contrast to the previous year score of -1.5.

Subsequently, a callback DXA scan was conducted a month later. It revealed a right femoral neck T-score of -1.3 and a left femoral neck T-score of -1.7, both were not significantly changed from the prior study. This prompted an investigation into the reasons behind the initial error in the left femoral neck T-score.

Despite utilizing scanners at two different locations from the same manufacturer, namely the Horizon Bone Densitometry System Apex, and ensuring identical equipment installation and maintenance procedures, no technical issues were identified upon reviewing data from 6 other patients who underwent DXA scans on the same day with the same scanner.

**Figure 1.**
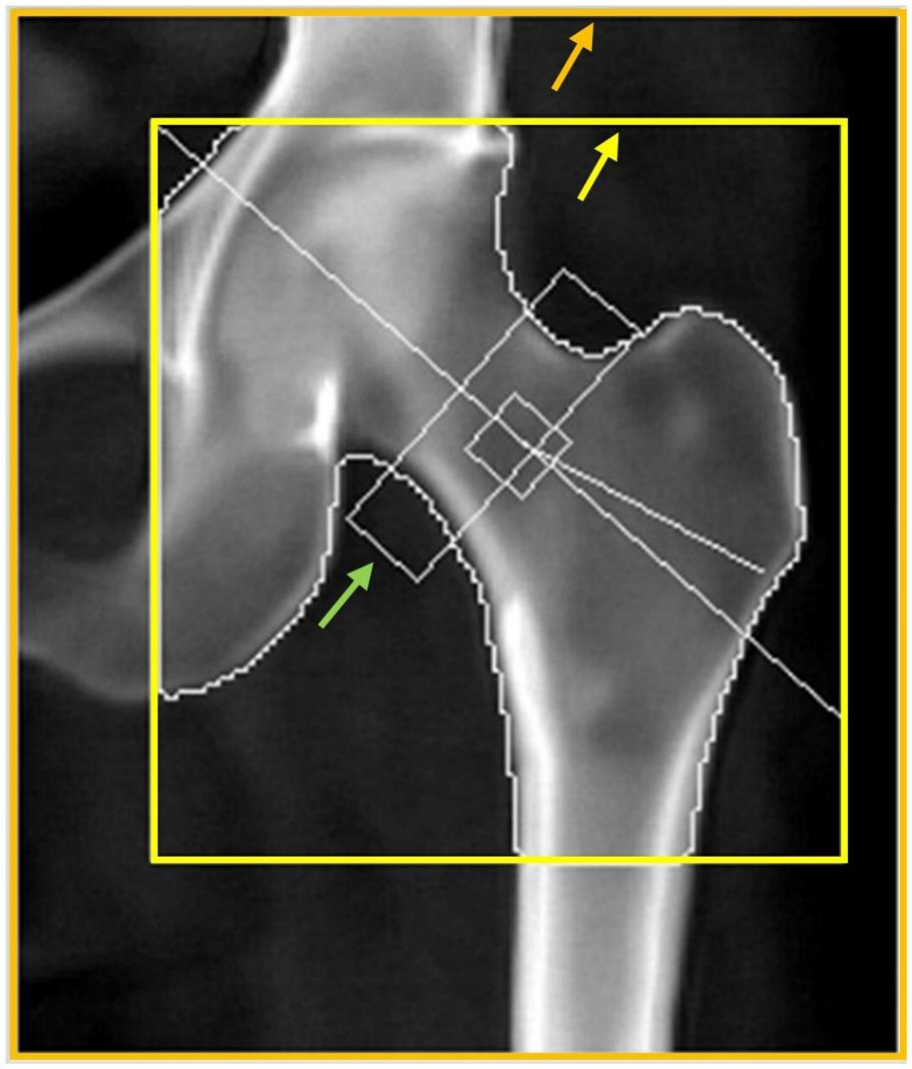
A Properly analyzed hip scan for this patient as a callback.

Further examination led to the discovery of a discrepancy in the region of interest positioning protocols outlined in the user guide of the Horizon system. Specifically, for hip measurements, the manual recommends placing the laser’s crosshair 7.6 cm below the greater trochanter and 2.5 cm medial to the femur’s shaft on the control panel. Proper adherence to this protocol, as illustrated in Figure 1, is essential for ensuring accurate analysis of hip scans.

As Figure 1 shows, the orange arrow frame indicates the field of view (FOV), with its lower edge ideally positioned at least 7.6 cm below the greater trochanter and the inner edge about 2.5 cm from the femur’s shaft, as per the user guide recommendations. The yellow arrow frame outlines the global region of interest (GROI), with its upper and inner edges aligned with the acetabulum. The rectangle that is represented using the green arrow denotes the neck region of interest, positioned vertically to the dashed oblique line and in close proximity to the greater trochanter.

**Figure 2.**
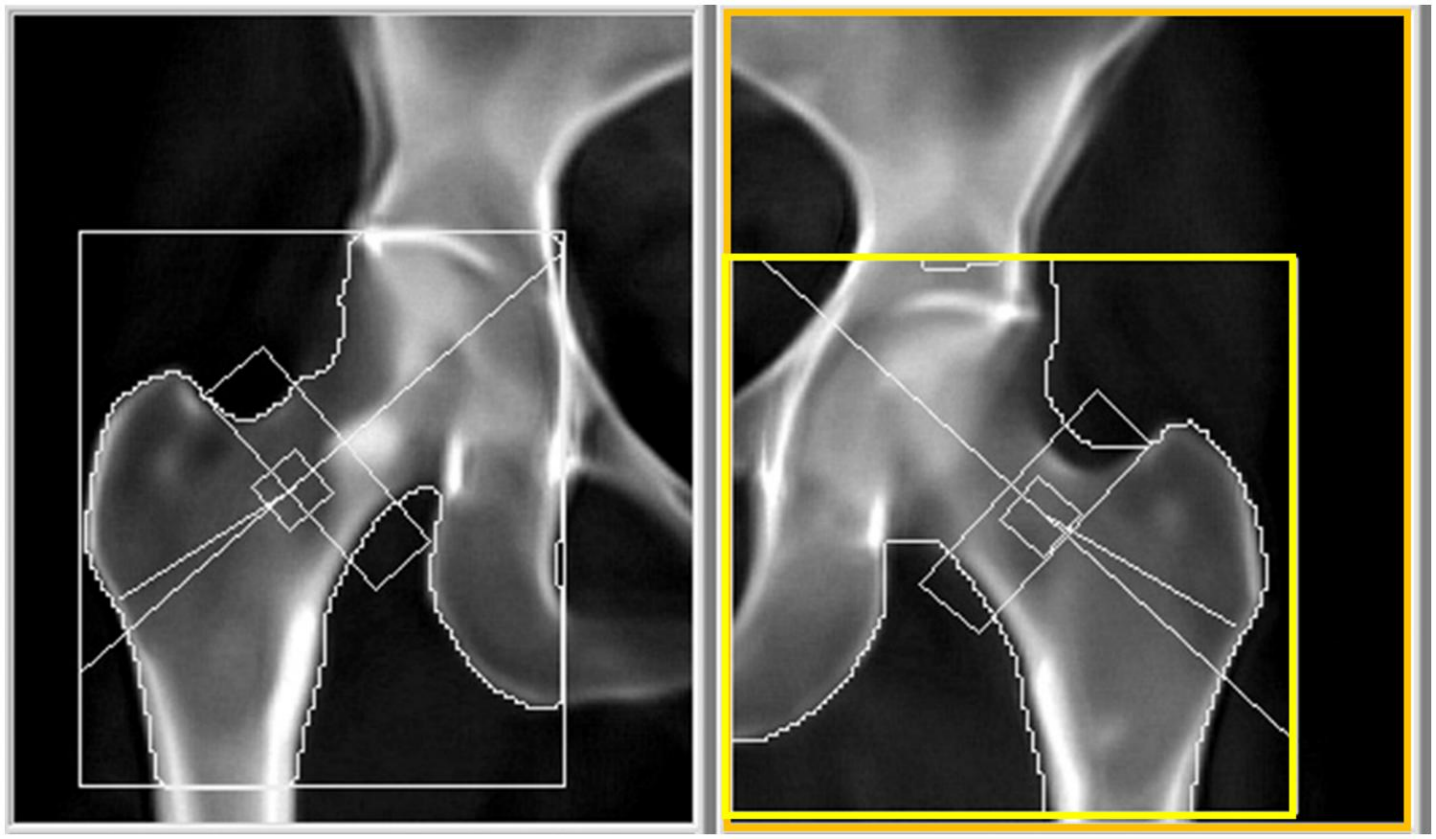
The initial DXA scan, the T-score of left femoral neck (right side) recorded as -6.8, while d_0_ was 270.3. DXA = dual-energy X-ray absorptiometry.

**Figure 3.**
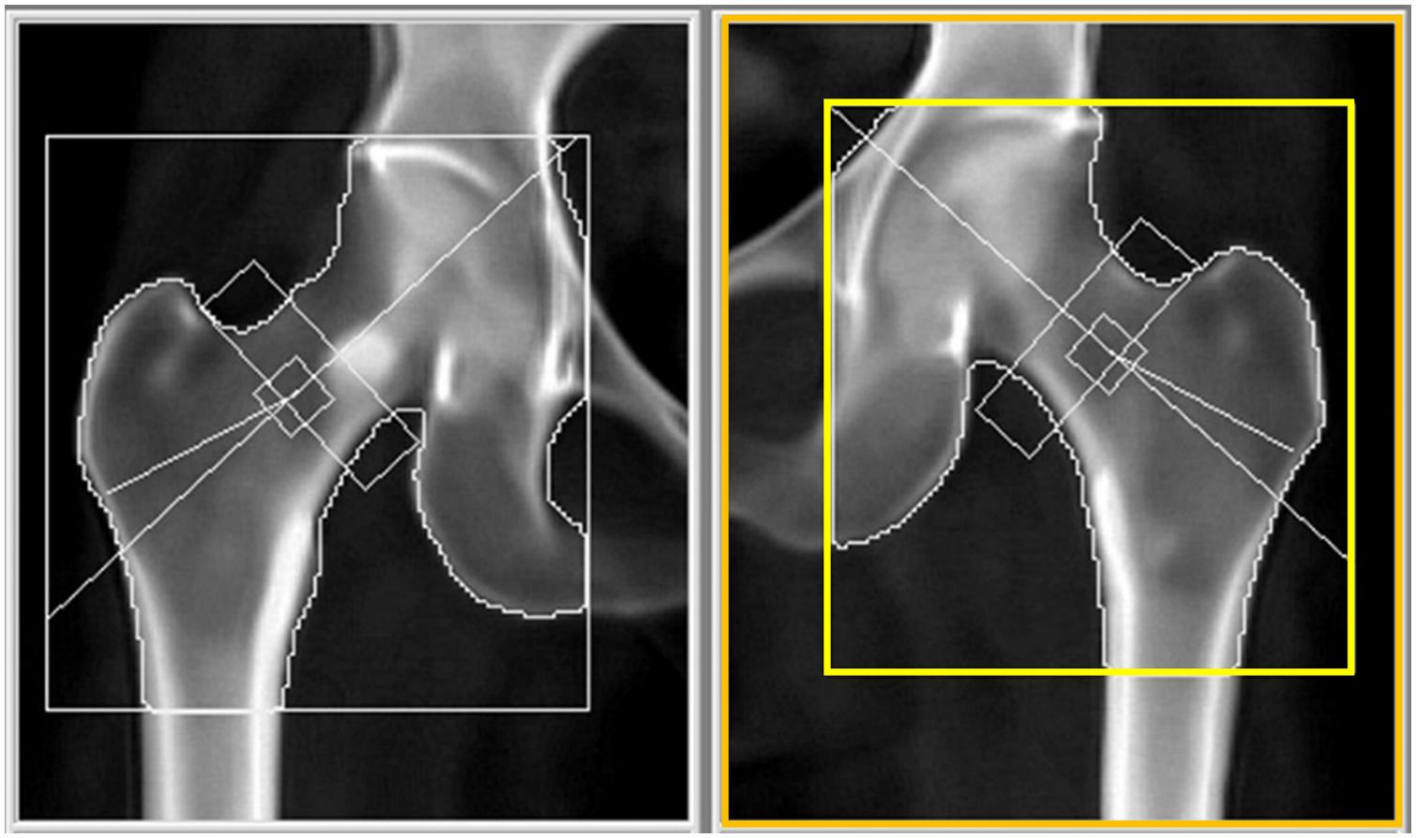
DXA scan conducted 1 month later as a callback, the T-score of left femoral neck recorded as -1.7, while d_0_ was 51.3. DXA = dual-energy X-ray absorptiometry.

In a Horizon DXA scan, “d_0_” refers to the calculated bone mineral density of a specific anatomical site. It represents the density of bone tissue at the reference point, often referred to as the “zero thickness” point, which serves as the baseline for comparison with the patient’s actual bone density measurements. No literature about d_0_ was found, so the authors checked 20 patients with correctly analysed DXA scan of the hip, and figured out the d_0_ usually falls into the range of 45-55, with average d_0_ around 50. The two DXA scans of this same patient were then compared in detail (Figures 2 and 3).

Upon examination of Figure 2, it was evident that the FOV (orange box) of the left hip is inadequately small, particularly noticeable in the lower margin, which measures only 4.25 cm below the greater trochanter, falling short of the recommended 7.6 cm. Additionally, the GROI (yellow box) was incorrectly positioned, as the upper and inner lines fail to be aligned to the acetabulum.

Figure 3 demonstrates a corrected left hip analysis, the lower margin of the FOV (orange box) measures about 8.2 cm below the greater trochanter, the GROI (yellow box) indicating appropriate adjustments made following the previous discrepancy.

Furthermore, re-processing of the initial DXA scan was conducted, incorporating several post-processing experiments to assess the impact of different sizes of the GROI box and various bone map segmentations on d_0_ and T-score values. The experimental results revealed significant fluctuations in d_0_ and T-score values based on the size of the GROI box, illustrated in Figure 1. Specifically, d_0_ ranged from 136.8 to 56.4, while T-score varied from -6.2 to -3.3. Additionally, for this particular case, concerning the left hip, the automatic bone map segmentation also did not accurately delineate the boundaries, encompassing part of the soft tissue above the femoral neck and greater trochanter while omitting a portion of the ischium bone. Subsequent manual adjustments, involving the deletion and addition of bone segments, led to a further increase in the T-score to -2.1 from -3.3.

**Figure 4.**
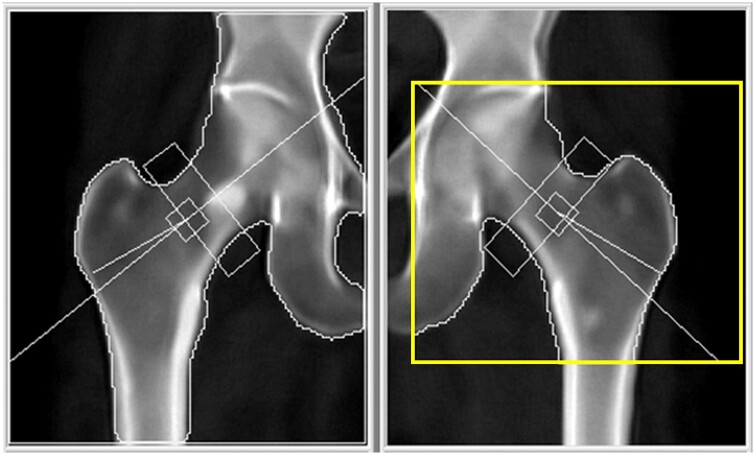
The initial DXA scan of 6-month follow up, the T-scores of the right and left femoral neck were recorded as -0.7 and -2.7, respectively; while d_0_ of the right hip was 56.1, left hip was 54.6. DXA = dual-energy X-ray absorptiometry.

The patient underwent another DXA follow-up examination 6 months later. Unexpectedly, the initial scan analysis once again failed to comply with the recommendations outlined in the user guide, particularly regarding the correct placement of the GROI (Figure 4).

Figure 4 demonstrates adequate FOV for both hips. However, the right GROI box was missing from the report, and the left GROI (yellow box) was improperly positioned, with its outer line too far from the greater trochanter. This resulted in T-scores of -0.7 for the right femoral neck and -2.7 for the left femoral neck.

**Figure 5.**
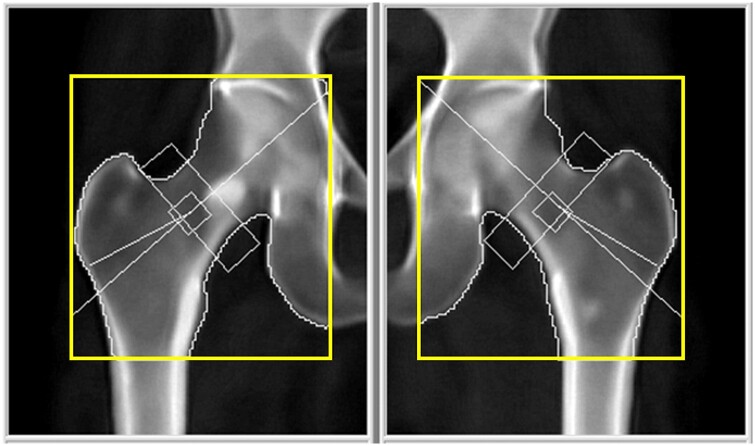
DXA scan of 6-month follow up that re-processed and analysed correctly, the T-scores of the right and left femoral neck were recorded as -1.5 and -2.0, respectively; while d_0_ of the right hip was 54.4, left hip was 53.4. DXA = dual-energy X-ray absorptiometry.

The left hip T-score of -2.7 represented a significant decrease compared to 6 months ago when it was -1.7 (as shown in Figure 3). Following this, a re-processing of the scan was conducted, ensuring the correct placement of the bilateral GROI (yellow boxes), as illustrated in Figure 5. As a result, the T-scores for the right and left femoral neck were determined to be -1.5 and -2.0, respectively, consistent with osteopenia.

## Discussion

DXA is often considered the gold standard imaging test to diagnose osteoporosis, but it has limitations.^1–3^ DXA acquisition and reporting errors are common.^4^ Errors can happen at every stage of DXA acquisition, analysis, interpretation, and quality control.^5^ One common error is from inadequate technician training and education, resulting in improper image acquisition and analysis.^2^^,^^5^ Inaccuracies in DXA report may lead to incorrect patient care decisions. This case emphasizes the vital importance of thorough technician training and strict adherence to manufacturer protocols in DXA scanning.

The initial scan’s inaccuracies resulted from inadequate FOV and misplacement of the GROI box and recurred in a follow-up DXA scan 6 months later, conducted by a different technologist at the same site. This suggests that insufficient technician training and education may be common within the institution. It is crucial for technologists to be able to identify and understand all items on the DXA report, including “d_0_.” This value can assist them in promptly recognizing inaccuracies in report analysis, and possibly directing them to proceed with re-processing to correct any error.

Experimental re-processing further emphasizes the impact of these deficiencies on diagnostic accuracy. Simply through re-processing the acquired images, the T-score can undergo dramatic changes, resulting in differing diagnoses. This significantly impacts the patient and subsequent clinical care. At times, manual adjustments to address segmentation errors are necessary, highlighting the importance of technologists’ vigilant oversight where automated processes fail.

In addition, the International Society for Clinical Densitometry provides guidance for best practices in DXA measurement and reporting, which both technologists and physicians should be familiar with.^6^ Research suggests that implementing a DXA reporting template reduces major errors and should become a common practice.^4^

This case serves as a warning sign during clinical practice, alerting both technologists and physicians to be aware of the common errors during DXA acquisition and interpretation. It emphasizes the critical need for continuous quality assurance and robust technician and physician education to ensure precise DXA assessments, which are crucial for effective patient care and address emerging challenges in DXA scanning.

## Conclusion

Technologists responsible for operating DXA equipment must undergo comprehensive training to ensure proficiency in scan acquisition and analysis. This training should encompass proper positioning techniques, adherence to manufacturer guidelines, and proficiency in performing consistent scans.

While DXA remains a cornerstone in bone health assessment, its effectiveness is contingent upon the proficiency of staff involved in scan acquisition and analysis. Investing in comprehensive staff training and education is paramount to minimizing errors, ensuring accurate DXA assessments, and ultimately improving patient outcomes in the diagnosis and management of osteoporosis and other skeletal disorders.

## Learning points

- Technologists and physicians should be aware of common errors in DXA acquisition and interpretation to recognize inaccuracies in a DXA report and take appropriate corrective actions.
- This case demonstrates pitfalls in post-processing of DXA analysis that can change the T-score significantly and highlights the importance of training for DXA technologists to adhere to manufacturers’ recommendations to ensure the accuracy, consistency, and reliability of DXA scans.

## References

[1] Link TM. Osteoporosis imaging: state of the art and advanced imaging. Radiology. 2012;263(1):3-17. 10.1148/radiol.2633201203 22438439 PMC3309802

[2] Guglielmi G , Diano D, Ponti F, Bazzocchi A. Quality assurance in bone densitometry. Curr Radiol Rep. 2014;2(2):33. 10.1007/s40134-013-0033-9

[3] Choksi P , Jepsen KJ, Clines GA. The challenges of diagnosing osteoporosis and the limitations of currently available tools. Clin Diabetes Endocrinol. 2018;4:12. 10.1186/s40842-018-0062-7 PMC5975657 29862042

[4] Krueger D , Shives E, Siglinsky E, et al DXA errors are common and reduced by use of a reporting template. J Clin Densitom. 2019;22(1):115-124. 10.1016/j.jocd.2018.07.014 30327243

[5] Lewiecki EM , Lane NE. Common mistakes in the clinical use of bone mineral density testing. Nat Clin Pract Rheumatol. 2008;4(12):667-674. 10.1038/ncprheum0928 18936788 PMC3891842

[6] Lewiecki EM , Binkley N, Morgan SL, et al; International Society for Clinical Densitometry. Best practices for dual-energy x-ray absorptiometry measurement and reporting: International Society for Clinical Densitometry Guidance. J Clin Densitom. 2016;19(2):127-140. 10.1016/j.jocd.2016.03.003 27020004

